# Smaller left ventricular end-systolic diameter and lower ejection fraction at baseline associated with greater ejection fraction improvement after revascularization among patients with left ventricular dysfunction

**DOI:** 10.3389/fcvm.2022.967039

**Published:** 2022-09-29

**Authors:** Shaoping Wang, Yi Lyu, Shujuan Cheng, Yuchao Zhang, Xiaoyan Gu, Ming Gong, Jinghua Liu

**Affiliations:** ^1^Department of Cardiology, Beijing Anzhen Hospital, Beijing Institute of Heart Lung and Blood Vessel Diseases, Capital Medical University, Beijing, China; ^2^Department of Anesthesiology, Minhang Hospital, Fudan University, Shanghai, China; ^3^Department of Echocardiography, Beijing Anzhen Hospital, Beijing Institute of Heart Lung and Blood Vessel Diseases, Capital Medical University, Beijing, China; ^4^Department of Cardiovascular Surgery, Beijing Anzhen Hospital, Beijing Institute of Heart Lung and Blood Vessel Diseases, Capital Medical University, Beijing, China

**Keywords:** ejection fraction, heart failure, remodeling, revascularization, prognosis

## Abstract

**Objectives:**

To investigate the predictive roles of pre-operative left ventricular (LV) size and ejection fraction (EF) in EF improvement and outcome following revascularization in patients with coronary artery disease (CAD) and LV dysfunction.

**Background:**

Revascularization may improve EF and long-term outcomes of patients with LV dysfunction. However, the determinants of EF improvement have not yet been investigated comprehensively.

**Materials and methods:**

Patients with EF measurements before and 3 months after revascularization were enrolled in a cohort study (No. ChiCTR2100044378). All patients had baseline EF ≤ 40%. EF improvement was defined as absolute increase in EF > 5%. According to LV end-systolic diameter (LVESD) (severely enlarged or not) and EF (≤35% or of 36–40%) at baseline, patients were categorized into four groups.

**Results:**

A total of 939 patients were identified. A total of 549 (58.5%) had EF improved. Both LVESD [odds ratio (OR) per 1 mm decrease, 1.05; 95% CI, 1.04–1.07; *P* < 0.001] and EF (OR per 1% decrease, 1.06; 95% CI, 1.03–1.10; *P* < 0.001) at baseline were predictive of EF improvement after revascularization. Patients with LVESD not severely enlarged and EF ≤ 35% had higher odds of being in the EF improved group in comparison with other three groups both in unadjusted and adjusted analysis (all *P* < 0.001). The median follow-up time was 3.5 years. Patients with LVESD not severely enlarged and EF ≤ 35% had significantly lower risk of all-cause death in comparison with patients with LVESD severely enlarged and EF ≤ 35% [hazard ratio (HR), 2.73; 95% CI, 1.28–5.82; *P* = 0.009], and tended to have lower risk in comparison with patients with LVESD severely enlarged and EF of 36–40% (HR, 2.00; 95% CI, 0.93–4.27; *P* = 0.074).

**Conclusion:**

Among CAD patients with reduced EF (≤ 40%) who underwent revascularization, smaller pre-operative LVESD and lower EF had greatest potential to have EF improvement and better outcome. Our findings imply the indication for revascularization in patients with LV dysfunction who presented with lower EF but smaller LV size.

## Introduction

Among patients with heart failure (HF) with reduced ejection fraction (EF), EF was an important treatment target, as higher EF was associated with lower risk of all-cause mortality (1–3). Improvements in EF were also associated with better outcomes and remained an important treatment goal (1, 2, 4). Revascularization among coronary artery disease (CAD) patients with left ventricular (LV) dysfunction including coronary artery bypass graft (CABG) and percutaneous coronary intervention (PCI) may increase blood supply of ischemic myocardium thus improve LV performance and long-term outcomes (5–9). However, not all patients with reduced EF had EF improvement after revascularization (10). We recently reported that revascularization among patients with an initial EF of 40% or less, 58.8% had EF improved (absolute increase in EF > 5%), but 41.2% patients remained EF unimproved (absolute increase in EF ≤ 5%) (1). On the other hand, revascularization in patients with LV dysfunction was associated with a greater overall risk for adverse periprocedural events compared with similar patients who had normal LV function (11, 12). Thus selection of a particular subset of patients who are associated with the greatest absolute benefit afforded through revascularization is of clinical implication.

Diabetes mellitus and no history of myocardial infarction (MI) had been shown to be predictive of EF improvement after coronary revascularization in our previous study (1). The multicenter Surgical Treatment for Ischemic Heart Failure (STICH) trial (13) compared the efficacy of medical therapy alone with that of medial therapy plus CABG in patients with LV dysfunction (EF ≤ 35%). By using the data of STICH trial, the predictive role of three factors, i.e., presence of 3-vessel CAD, EF below the median (27%), and end-systolic volume index above the median (79 ml/m^2^) in outcomes after treatment were investigated (14). The study showed that a net beneficial effect of CABG relative to optimal medical therapy alone was observed in patients with 2–3 factors but not in those with 0–1 factor. The predictive role of pre-operative LV size and function among patients with reduced EF is still unclear. The best indication for revascularization in patients with LV dysfunction has not been well-established.

Therefore, this study was conducted to clarify (1) the predictive role of pre-operative LV end-systolic diameter (LVESD) and pre-operative EF in EF improvement following revascularization; and (2) outcomes difference among patients grouped by LVESD severely enlarged or not, and EF ≤ 35% or EF within 36–40%.

## Materials and methods

### Patient selection

This was a real-world cohort study conducted at the Beijing Anzhen Hospital. The study has been registered in Chinese Clinical Trial Registry (No. ChiCTR2100044378). The study protocol was approved by the hospital’s ethics committee. Individual patient consent was waived as this was a retrospective analysis of de-identified data in our institutional database.

Coronary artery disease Patients were enrolled if they had initial reduced EF (≤40%), and underwent isolated CABG or PCI, and had repeated echocardiographic measurements during follow-up (Supplementary Figure 1). Patients were excluded if they were diagnosed as ST-segment elevation MI, died within 3 months after revascularization, and had only one record of echocardiographic reassessment within 3 months after CABG or PCI. According to the absolute change in EF, patients were categorized: (1) EF unimproved group (absolute increase in EF ≤ 5%); and (2) EF improved group (absolute increase in EF > 5%) (15). Baseline LVESD severely enlarged was defined as LVESD ≥ 42 mm for women and ≥46 mm for men (16). Patients were further categorized: (1) LVESD not severely enlarged and EF ≤ 35% at baseline; (2) LVESD not severely enlarged and EF of 36–40%; (3) LVESD severely enlarged and EF ≤ 35%; and (4) LVESD severely enlarged and EF of 36–40%.

### Data collection and definitions

The clinical, laboratory, echocardiographic data and medical therapy were recorded from hospital medical records. Baseline echocardiographic data was captured within 30 days before PCI or CABG. Follow-up echocardiographic data were defined as the first measurement 3 months (17) after revascularization assessed in Beijing Anzhen Hospital. Complete revascularization was defined as successful PCI (residual stenosis of <30%) of all angiographically significant lesions (≥70% diameter stenosis) in 3 coronary arteries and their major branches. For CABG, grafting of every primary coronary artery with ≥70% diameter stenosis was accepted as complete revascularization.

Outcome data were obtained from medical records at Beijing Anzhen Hospital and through telephone follow up. The follow-up time for patients started at the time of the first available EF measurement (15, 18, 19).

### Statistical analysis

The categorical variables were reported as counts and percentages. Continuous variables were presented as mean ± standard deviation (SD) or median (interquartile range). Among 4 categories of patients, i.e., LVESD not severely enlarged and EF ≤ 35%, LVESD not severely enlarged and EF of 36–40%, LVESD severely enlarged and EF ≤ 35%, LVESD severely enlarged and EF of 36–40%, continuous variables were compared by ANOVA or a Kruskal–Wallis test. Tukey’s *post-hoc* test was used for pairwise comparisons of means with equal variances. Logistic regression was used to identify the predictive role of pre-operative LVESD and EF in EF improvement after revascularization. Odds ratios (OR) of being in the EF improved group were compared between 4 groups, and were adjusted with age, sex, body mass index, hypertension, status of diabetes, history of MI, severity of mitral regurgitation, treatment with PCI or CABG, and complete revascularization. The risks of outcomes were analyzed with a Cox proportional hazards regression model. In adjusted model, hazard ratio (HR) was adjusted by age, sex, body mass index, hypertension, renal function, chronic obstructive pulmonary disease, diabetic status, history of MI, severity of mitral regurgitation (MR), treatment with PCI or CABG, and complete revascularization. Kaplan–Meier survival curves were constructed to compare 4 categories of patients. All statistical analyses were based on 2-tailed tests. *P* < 0.05 was considered statistically significant. Statistical analyses were performed with Stata version 14.0 (StataCorp).

## Results

### Predictive roles of left ventricular end-systolic diameter and ejection fraction

Among 1,781 initially identified patients, 78 patients who died within 3 months after revascularization, 764 patients were further excluded because echocardiography was not evaluated 3 months after revascularization. Finally, 939 patients who had an initial EF ≤ 40% and had echocardiography reassessment 3 months after revascularization were enrolled in this study (Supplementary Figure 1). Of 1,878 person-time EF measurements, 928 (98.8%) EF measurements before revascularization and 872 (92.9%) EF measurements during follow-up were by Simpson.

The average age at baseline was 64.7 ± 10.8 years. Men comprised 83.5% of all subjects. A total of 533 (56.8%) received PCI and 406 (43.2%) underwent CABG. Median LVESD was 46 mm (interquartile range 40 mm to 52 mm) and median EF was 38% (interquartile range 35–40%). The mean age and distribution of sex were similar for PCI and CABG patients. The prevalence of hypertension, chronic kidney disease and diabetes mellitus and history of MI were also similar for PCI and CABG patients. The PCI patients had lower pre-operative EF (36.0 vs. 36.6%, *P* = 0.035) but similar LVESD (45.6 vs. 46.5 mm, *P* = 0.099) in comparison to CABG patients. Patients who underwent PCI had a lower prevalence of coronary multivessel disease (67.7 vs. 93.8%, *P* < 0.001) and a lower percentage of complete revascularization (40.2 vs. 73.4%, *P* < 0.001).

**FIGURE 1:**
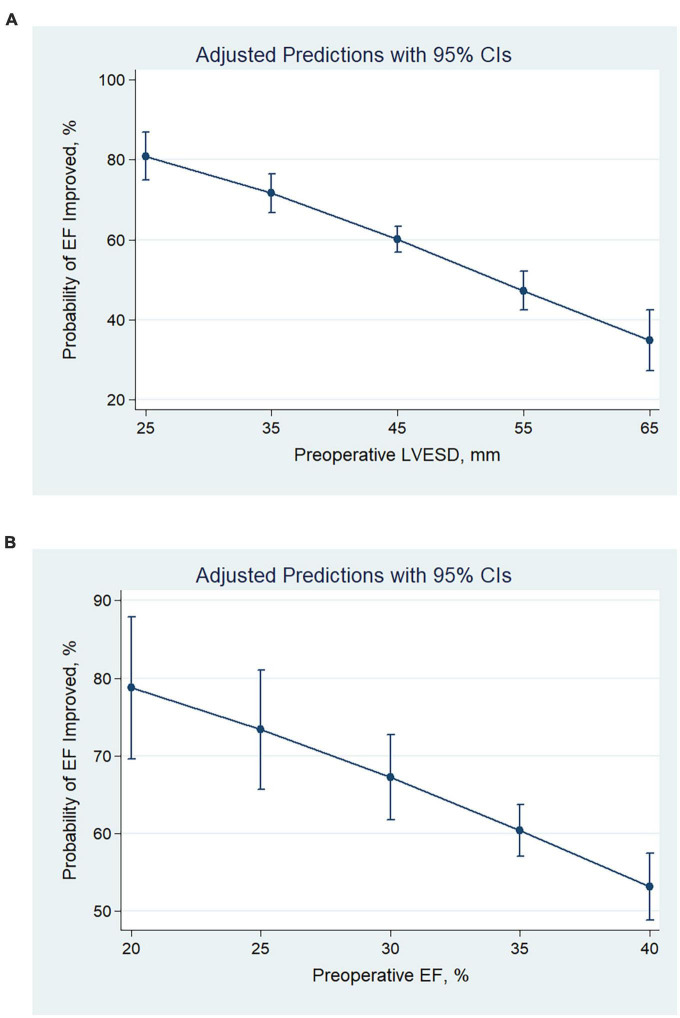
Predicted probabilities and the 95% confidence interval of EF improvement after revascularization at different values of pre-operative LVESD **(A)** and EF **(B)**. With the decrease in pre-operative LVESD or EF, the probability of EF improvement increased. CI, confidence interval; LVESD, left ventricular end-systolic diameter; EF, ejection fraction.

After revascularization, 549 (58.5%) had EF improved (absolute increase in EF > 5%), 390 (41.5%) had EF unimproved (absolute increase in EF ≤ 5%). Patients with smaller LV size had greater odds of being in the EF improved group (OR per 1 mm decrease, 1.05; 95% CI, 1.04–1.07; *P* < 0.001) (Figure 1A). Patients with lower pre-operative EF had higher odds of being in the EF improved group (OR per 1% decrease, 1.06; 95% CI, 1.03–1.10; *P* < 0.001) (Figure 1B).

**FIGURE 2:**
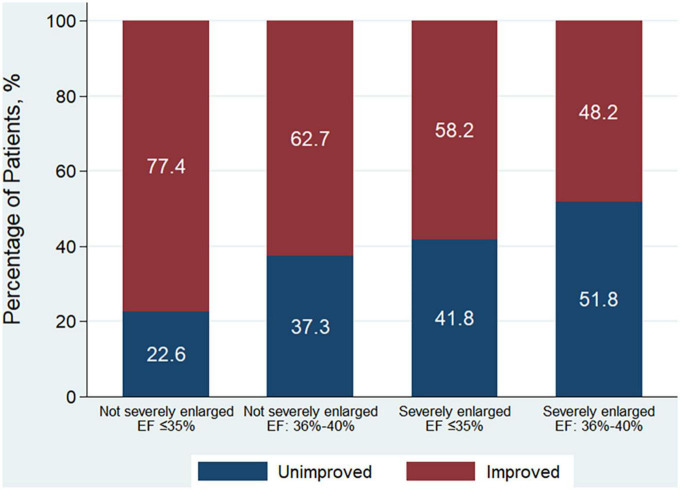
Patient distribution according to the improvement of EF among 4 patient groups according to whether LVESD was severely enlarged or not, and EF ≤ 35% or of 36–40% at baseline. EF improvement was defined as absolute increase in EF > 5% following revascularization. Men with LVESD ≥ 46 mm and women with LVESD ≥ 42 mm were defined as severely enlarged LVESD. LVESD, left ventricular end-systolic diameter; EF, ejection fraction.

Patients were further categorized into 4 groups according to whether LVESD was severely enlarged or not, and EF ≤ 35% or of 36–40%. Patients with LVESD not severely enlarged and EF ≤ 35% had higher odds of being in the EF improved group in comparison with other 3 groups both in unadjusted and adjusted analysis (all *P* < 0.001) (Table 1). A total of 77.4% of patients with LVESD not severely enlarged and EF ≤ 35% had EF improvement after revascularization, which was greatest in 4 groups (*P* < 0.001) (Figure 2).

**TABLE 1:** Odds ratio of EF improvement according to LVESD and EF at baseline.

	LVESD not severely enlarged EF ≤ 35%	LVESD not severely enlarged EF ≤ 36–40%	LVESD severely enlarged EF ≤ 35%	LVESD severely enlarged EF ≤ 36–40%
Unadjusted	Reference	0.49 (0.29–0.82)	0.41 (0.24–0.69)	0.27 (0.16–0.45)
Adjusted^a^	Reference	0.48 (0.28–0.81)	0.41 (0.24–0.71)	0.27 (0.16–0.46)

Data are OR (95% CI). Women with LVESD ≥ 42 mm and men with LVESD ≥ 46 mm were defined as severely enlarged LVESD. ^*a*^ORs were adjusted with age, sex, body mass index, status of diabetes, history of myocardial infarction, treatment with PCI or CABG, and complete revascularization. EF, ejection fraction; LVESD, left ventricular end-systolic diameter; OR, odds ratio.

### Characteristics of patients according to pre-operative left ventricular end-systolic diameter and ejection fraction

**FIGURE 3:**
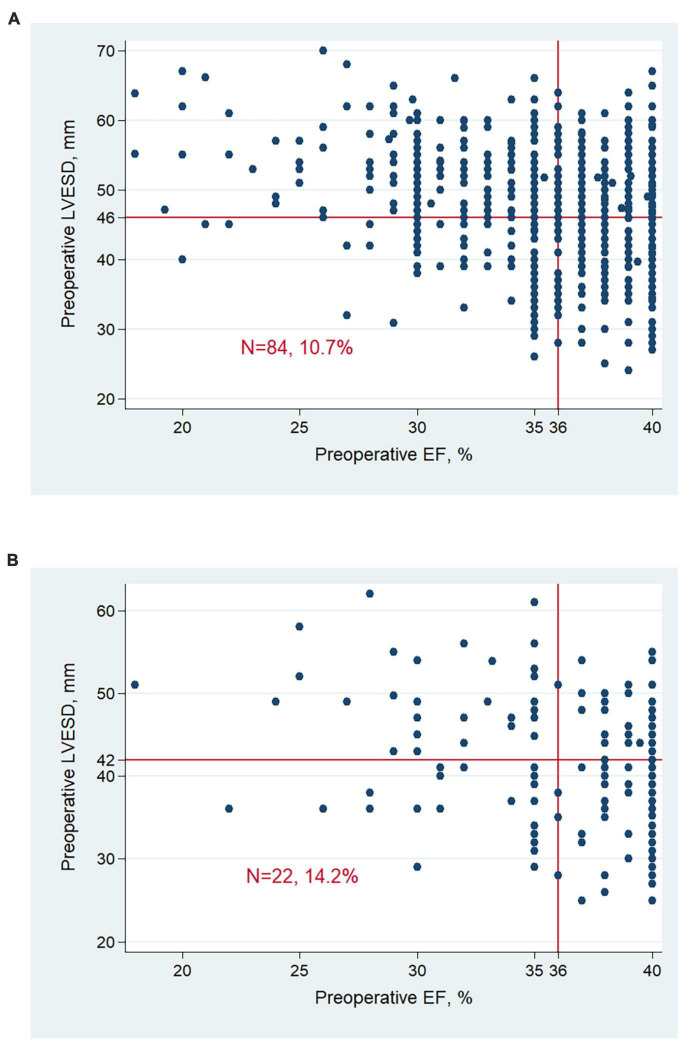
Patient distribution according to LVESD and EF at baseline in men **(A)** and women **(B)**. Men with LVESD ≥ 46 mm and women with LVESD ≥ 42 mm were defined as severely enlarged LVESD. LVESD, left ventricular end-systolic diameter; EF, ejection fraction.

Characteristics of patients were further investigated between 4 groups (Table 2). Patients’ distribution according to pre-operative LVESD and EF was indicated in Figure 3. A total of 84 (10.7%) men and 22 (14.2%) women had LVESD not severely enlarged and EF ≤ 35%. Demographics and medical history including DM and MI, were similar between 4 groups. There were significant differences of pre-operative EF, LVEDD, LVESD and presence of moderate to severe MR (*P* < 0.001). Furthermore, the difference of pre-operative EF between any 2 groups was significant (all *P* < 0.001) except for the comparison between 2 groups with EF of 36–40% (*P* = 0.224). After revascularization, patients with LVESD not severely enlarged and EF ≤ 35% had EF improvement of 14.6 ± 10.9%, which was greater than any other group (all *P* < 0.001). Post-operative EF, LVESD, and LVEDD between 2 groups with LVESD not severely enlarged were similar. However, post-operative EF, LVESD, and LVEDD in 2 groups with LVESD not severely enlarged were better than those in other 2 groups with LVESD severely enlarged, respectively (all *P* < 0.05). In addition, the difference of post-operative EF between any 2 groups was significant (all *P* < 0.05) except for the comparison between 2 groups with LVESD not severely enlarged (*P* = 0.282).

**TABLE 2:** Patient characteristics according to LVESD and EF at baseline^a^.

Characteristic	not severely enlarged EF ≤ 35%	not severely enlarged EF 36–40%	severely enlarged EF ≤ 35%	severely enlarged EF 36–40%	*P*-value
Patient number	106	300	220	313	
**Demographics and history**					
Age, year	65.4 (10.5)	63.8 (11.4)	65.1 (10.9)	65.0 (10.2)	0.344
Male sex	84 (79.3)	242 (80.7)	189 (85.9)	269 (85.9)	0.143
Body mass index	25.0 (4.9)	25.2 (4.1)	25.0 (3.7)	25.7 (4.2)	0.209
Current smoker	38 (35.9)	112 (37.3)	81 (36.8)	104 (33.2)	0.728
Hypertension	53 (50.0)	163 (54.3)	111 (50.5)	180 (57.5)	0.339
Chronic kidney disease	15 (14.2)	39 (13.0)	27 (12.3)	46 (14.7)	0.857
DM	36 (34.0)	105 (35.0)	80 (36.4)	103 (32.9)	0.865
COPD	7 (6.7)	11 (3.7)	4 (1.9)	20 (6.5)	0.051
Prior stroke	10 (9.4)	16 (5.3)	23 (10.5)	19 (6.1)	0.093
Atrial fibrillation	3 (2.8)	19 (6.3)	9 (4.1)	12 (3.8)	0.338
History of MI	42 (39.6)	133 (44.3)	113 (51.4)	151 (48.2)	0.170
History of PCI	20 (18.9)	52 (17.3)	36 (16.4)	62 (19.8)	0.747
History of CABG	1 (0.94)	5 (1.7)	11 (5.0)	10 (3.2)	0.098
**Echocardiography**					
**Pre-operative**					
EF, %	32.6 (3.3)	39.0 (1.4)	30.9 (3.9)	38.6 (1.4)	<0.001
LVEDD, mm	53.5 (5.2)	52.3 (5.0)	64.0 (5.5)	62.6 (5.3)	<0.001
LVESD, mm	39.3 (4.7)	38.4 (5.0)	53.2 (5.2)	50.5 (4.5)	<0.001
MR (moderate or severe)	18 (17.0)	25 (8.3)	58 (26.4)	62 (19.8)	<0.001
Post-operative					
EF, %	47.2 (10.9)	49.3 (10.8)	39.5 (10.9)	44.3 (10.1)	<0.001
LVEDD, mm	54.6 (7.0)	53.4 (6.7)	61.7 (9.0)	59.8 (8.1)	<0.001
LVESD, mm	39.7 (7.7)	38.3 (7.4)	48.6 (11.0)	46.0 (9.1)	<0.001
MR (moderate or severe)	12 (11.3)	27 (9.0)	53 (24.1)	50 (16.0)	<0.001
Change of EF, %	14.6 (10.9)	10.3 (10.8)	8.6 (11.0)	5.6 (10.0)	<0.001
Change of LVEDD, mm	1.0 (7.0)	1.2 (6.0)	−2.3 (7.7)	−2.7 (7.2)	<0.001
Change of LVESD, mm	0.3 (7.7)	−0.1 (6.9)	−4.6 (9.8)	−4.5 (8.4)	<0.001
**Angiography and therapy**					
Multi-vessel disease	77 (72.6)	229 (76.3)	182 (82.7)	254 (81.2)	0.086
Left main disease	7 (6.6)	22 (7.3)	12 (5.5)	15 (4.8)	0.583
PCI	64 (60.4)	183 (61.0)	128 (58.2)	158 (50.5)	0.047
CABG	42 (39.6)	117 (39.0)	92 (41.8)	115 (49.5)	0.047
Complete revascularization	67 (63.2)	156 (52.0)	113 (51.4)	176 (56.2)	0.155
ACEi/ARB/ARNI	51 (48.1)	153 (51.0)	113 (51.4)	159 (50.8)	0.953
β-Blocker	87 (82.1)	249 (83.0)	164 (74.6)	258 (82.4)	0.069
MRA	21 (19.8)	42 (14.0)	47 (21.4)	59 (18.9)	0.149

^a^Values are mean (SD) or No. of patients (%). ACEi, angiotensin-converting enzyme inhibitor; ARB, angiotensin receptor blocker; ARNI, angiotensin receptor-neprilysin inhibitor; CABG, coronary artery bypass grafting; COPD, chronic obstructive pulmonary disease; DM, diabetes mellitus; eGFR, estimated glomerular filtration rate; EF, ejection fraction; LVEDD, left ventricular end-diastolic diameter; LVESD, left ventricular end-systolic diameter; MI, myocardial infarction; PCI, percutaneous coronary intervention; MR, mitral regurgitation; MRA, mineralocorticoid receptor antagonist.

In addition, patients with LVESD not severely enlarged tended to have lower prevalence of multi-vessel diseases (*P* = 0.086) and received higher proportion of revascularization treatment with PCI (*P* = 0.047). Percentages of complete revascularization between 4 groups were similar (*P* = 0.155).

### Outcomes of patients according to pre-operative left ventricular end-systolic diameter and ejection fraction

**FIGURE 4:**
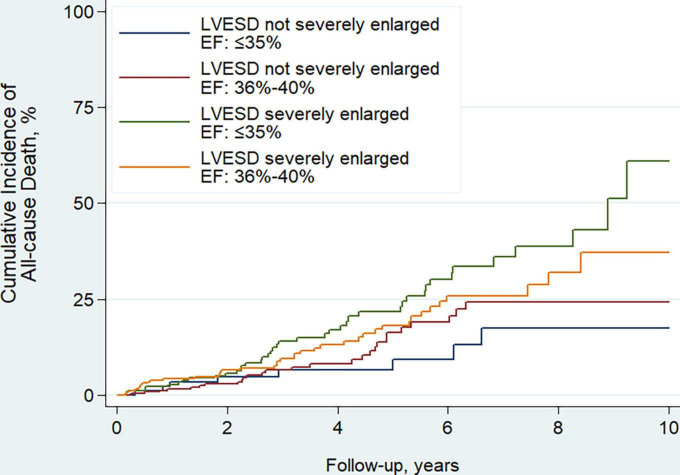
Kaplan–Meier curves estimating incidence of all-cause death after revascularization among 4 patient groups according to whether LVESD was severely enlarged or not, and EF ≤ 35% or of 36–40% at baseline. Men with LVESD ≥ 46 mm and women with LVESD ≥ 42 mm were defined as severely enlarged LVESD. LVESD, left ventricular end-systolic diameter; EF, ejection fraction.

The median follow-up time was 3.5 years, during which 137 patients died. Of those, 7 (5.1%) died of MI, 44 (32.1%) died of HF, 60 (43.8%) died suddenly, 1 (0.7%) did of other cardiac causes, and 25 (18.2%) died of non-cardiac causes. EF improvement was associated with lower risk of all-cause death (HR, 0.45; 95% CI, 0.31–0.65; *P* < 0.001). Compared to patients with LVESD not severely enlarged and EF ≤ 35%, patients with LVESD severely enlarged and EF ≤ 35% had significantly higher risk of all-cause death (HR, 2.73; 95% CI, 1.28–5.82; *P* = 0.009), and patients with LVESD severely enlarged and EF of 36–40% tended to have higher risk of all-cause death (HR, 2.00; 95% CI, 0.93–4.27; *P* = 0.074) (Table 3 and Figure 4). Two groups of patients with LVESD not severely enlarged had similar risk of mortality (HR, 1.45; 95% CI, 0.66–3.20; *P* = 0.356). These results persisted in adjusted models.

**TABLE 3:** Risk of all-cause death.

Outcomes	Unadjusted HR (95% CI)	*P*-Value	Adjusted HR (95% CI)	*P*-Value
LVESD not severely enlarged EF ≤ 35%	Reference		Reference	
LVESD not severely enlarged EF: 36–40%	1.45 (0.66–3.20)	0.356	1.44 (0.62–3.36)	0.400
LVESD severely enlarged EF ≤ 35%	2.73 (1.28–5.82)	0.009	2.34 (1.04–5.29)	0.040
LVESD severely enlarged EF: 36–40%	2.00 (0.93–4.27)	0.074	1.86 (0.82–4.19)	0.135

EF, ejection fraction; LVESD, left ventricular end-systolic diameter; HR, hazard ratio; Ref, reference. Women with LVESD ≥ 42 mm and men with LVESD ≥ 46 mm were defined as severely enlarged LVESD. HR was adjusted by age, sex, body mass index, hypertension, renal function, chronic obstructive pulmonary disease, diabetic status, history of myocardial infarction, severity of mitral regurgitation, treatment with PCI or CABG, and complete revascularization.

In addition, to clarify the short-term outcomes, the mortalities within 3 months after revascularization among four groups were compared. Of 78 patients who died within 3 months, 6 (7.7%) died of MI, 44 (56.4%) died of HF, 13 (16.7%) died suddenly, 5 (6.4%) did of other cardiac causes, and 10 (12.8%) died of non-cardiac causes. Compared to patients with LVESD not severely enlarged and EF ≤ 35%, patients with LVESD not severely enlarged and EF of 36–40% (OR, 1.10; 95% CI, 0.50–2.41; *P* = 0.809), patients with LVESD severely enlarged and EF ≤ 35% (OR, 1.14; 95% CI, 0.51–2.52; *P* = 0.750), and patients with LVESD severely enlarged and EF of 36–40% (OR, 0.88; 95% CI, 0.40–1.94; *P* = 0.748) had similar risk of mortality. These results persisted in adjusted models.

## Discussion

In the present study of CAD patients with reduced EF (≤40%), we found that (1) both smaller pre-operative LVESD and lower pre-operative EF had predictive roles of EF improvement after revascularization; (2) Patients with LVESD not severely enlarged and EF ≤ 35% at baseline had greatest EF improvement; and (3) Patients with LVESD not severely enlarged and EF ≤ 35% had better outcome of all-cause death after revascularization.

Improvements in EF were associated with better outcomes and remained an important treatment goal (1, 2, 4). CHAMP-HF (Change the Management of Patients with Heart Failure) was a prospective registry of outpatients with HF with reduced EF (20). In this study, 40% had an ischemic cardiomyopathy, and median EF at baseline was 30%. After appropriate treatment, 49% had a ≥5% increase in EF. Non-ischemic cardiomyopathy, no coronary disease, lower baseline EF, shorter HF duration and no implantable cardioverter defibrillator were associated with ≥5% EF increase. In another study enrolled patients with newly documented EF ≤ 35%, 46% had reverse remodeling which was defined as an improvement in EF to >35% at follow-up after 3 months (21). The baseline LVESD index, female gender and non-ischemic cardiomyopathy were independent predictors of LV reverse remodeling. However, baseline EF was not an independent predictor. In the current study, all patients were diagnosed as ischemic cardiomyopathy and received revascularization therapy. Men comprised 83.5% of all subjects due to sex difference in the prevalence of CAD. A total of 58.5% had EF improvement (absolute increase in EF > 5%) after revascularization. Smaller pre-operative LVESD and lower EF were associated with greater EF improvement after revascularization. Furthermore, patients with pre-operative LVESD not severely enlarged and EF ≤ 35% had greatest potential to have EF improvement after revascularization. As HF was heterogeneous in both etiology and pathophysiology as well as treatment, Our finding might reveal more precise correlates associated with EF improvement in ischemic cardiomyopathy after revascularization.

The predictive role of LV size and function in outcomes of patients with LV dysfunction remains controversial (3, 4, 14, 22). Study using echocardiographic data of Valsartan Heart Failure Trial (Val-HeFT) investigated the benefits of valsartan treatment in patients with reduced EF (<40%) (3). The study reported that both smaller baseline LVESD and higher EF were independently associated lower risk of mortality. In STICH trial, (14) patients with 2–3 factors, i.e., presence of 3-vessel CAD, EF < 27%, and LV end-systolic volume index >79 ml/m^2^ had higher risk of mortality in medical treatment only group, but statistically similar risk in CABG group, in comparison with patients with 0–1 factors. In the current study, patients with LVESD not severely enlarged and EF ≤ 35% had similar risk of death within 3 months after revascularization, but had better outcome of all-cause mortality during long-term follow-up. The risk reduction might be associated with greater EF improvement after revascularization between four groups. The predictive roles of LV size and EF in outcomes of HF patients with distinct etiology need to be further investigated.

In the current study, we identified a special patient cohort with pre-operative LVESD not severely enlarged and EF ≤ 35%, which had greatest potential to have EF improvement after revascularization and better outcome of mortality. This cohort accounted for about 10% of all study patients. It is unclear whether low LV function but without severely structure dilation represents a stage during LV remodeling or it could be the result of underlying molecular events involved in remodeling (23). In clinical practice, identification of patients with LVESD not severely enlarged and low baseline EF before revascularization might contribute to determine the best indication of revascularization among HF patients with reduced EF. On the contrary, patients with LVESD severely enlarged and low EF had worse outcome. Close care and follow-up were required to improve their expected poor outcome.

In the current study, pre-operative EF of 35%, LVESD ≥ 42 mm for women and ≥46 mm for men, (16) were used to categorize the patient groups artificially. The optimal values of EF and LVESD with better predictable role are required to be investigated in the future prospective study with large samples. In addition, all patients in the current study underwent isolated CABG. Moderate to severe mitral regurgitation were not treated simultaneously. However, mitral regurgitation might have a great impact on cardiac function recovery and outcomes (24). This needs to be further investigated.

## Limitations

Our study had several limitations. First, this observational study from a single center may have inherent selection bias. Second, echocardiographic evaluation will be affected by intra- and inter-observer variability. In this study, patients who did not have echocardiography reassessment 3 months after revascularization in one hospital were excluded. This increased the number of excluded patients but decreased the variability of EF measurement. Third, the EF value and mitral insufficiency are associated with patient’s loading conditions, which are not represented in current study. Fourth, we highlighted that in outcome analysis, risk of mortality was evaluated among patients who are survivors 3 months at least after revascularization.

## Conclusion

Among CAD patients with reduced EF (≤40%), smaller pre-operative LVESD and lower EF predicted greater EF improvement after revascularization. Patients with LVESD not severely enlarged and EF ≤ 35% had greatest potential to have EF improvement after revascularization and better outcome of mortality. Our findings imply the indication for revascularization in patients with LV dysfunction who presented with lower EF but smaller LV size. Special care after revascularization should be provided to patients who presented with lower EF and severely enlarged LV.

## Data availability statement

The raw data supporting the conclusions of this article will be made available by the authors, without undue reservation.

## Ethics statement

The studies involving human participants were reviewed and approved by the Ethics Committee of Beijing Anzhen Hospital. Written informed consent for participation was not required for this study in accordance with the national legislation and the institutional requirements.

## Author contributions

JL conceived the concept of the study and supervision. SW contributed to the design of the research. SW and YL wrote the original-draft. All authors involved in data collection and analysis, edited, and approved the final version of the manuscript.

## Supplementary material

Supplementary Figure 1 Patients selection and group procedure. Men with LVESD ≥ 46 mm and women with LVESD ≥ 42 mm were defined as severely enlarged LVESD. CABG, coronary artery bypass grafting; PCI, percutaneous coronary intervention; Echo, echocardiograph; EF, ejection fraction; LVESD, left ventricular end-systolic diameter.
