# Supplementary figures and images for: Smaller left ventricular end-systolic diameter and lower ejection fraction at baseline associated with greater ejection fraction improvement after revascularization among patients with left ventricular dysfunction
Source: Front Cardiovasc Med. 2022 Sep 29;9:967039. doi: 10.3389/fcvm.2022.967039 (PMC9559822; doi:10.3389/fcvm.2022.967039)

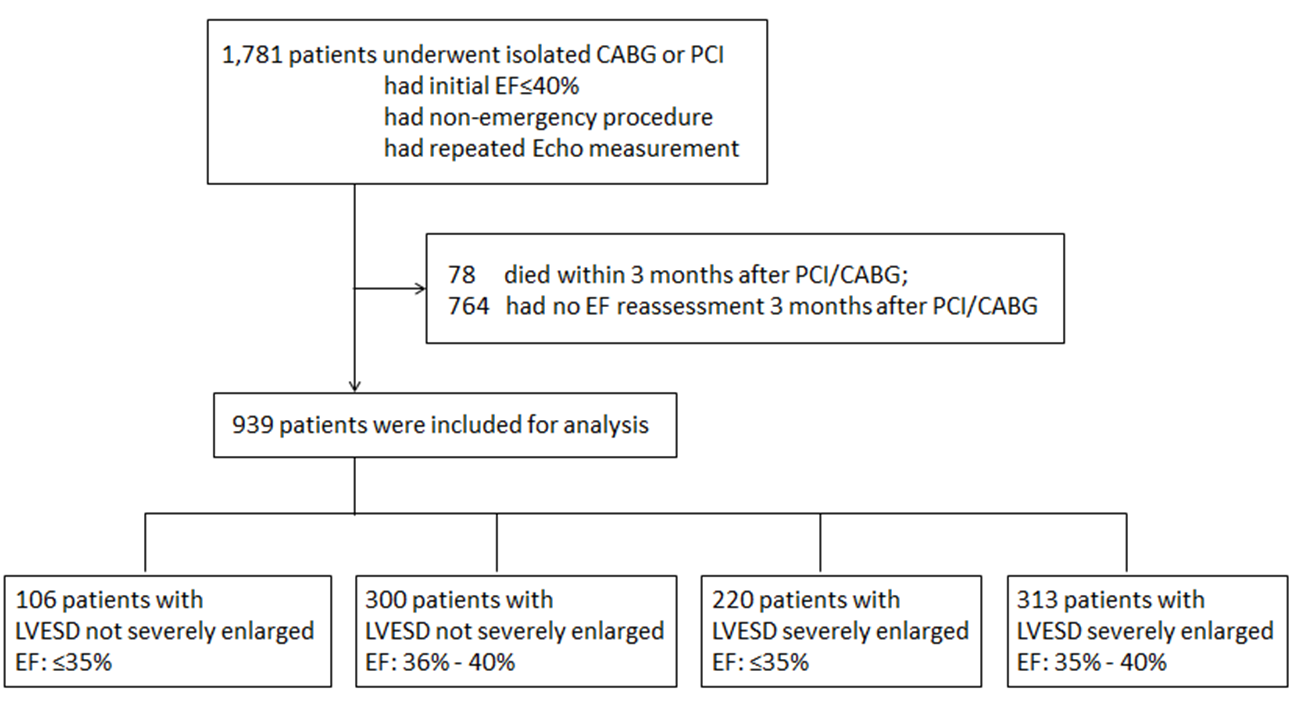

Supplement: Supplementary Figure 1 — Patients selection and group procedure. Men with LVESD ≥ 46 mm and women with LVESD ≥ 42 mm were defined as severely enlarged LVESD. CABG, coronary artery bypass grafting; PCI, percutaneous coronary intervention; Echo, echocardiograph; EF, ejection fraction; LVESD, left ventricular end-systolic diameter. [file Image_1.TIF]
